# Effect of the delegation of GP-home visits on the development of the number of patients in an ambulatory healthcare centre in Germany

**DOI:** 10.1186/1472-6963-12-355

**Published:** 2012-10-10

**Authors:** Neeltje van den Berg, Romy Heymann, Claudia Meinke, Sebastian E Baumeister, Steffen Fleßa, Wolfgang Hoffmann

**Affiliations:** 1 University Medicine Greifswald, Institute for Community Medicine, Ellernholzstr. 1-2, 17487, Greifswald, Germany; 2 University of Duisburg-Essen, Institute for Health Care Management and Research, Schützenbahn 70, 45127, Essen, Germany; 3 University Medicine Greifswald, Institute for Community Medicine, Walter-Rathenau-Str. 48, 17475, Greifswald, Germany; 4 University of Greifswald, Faculty of Law and Economics, Friedrich-Loeffler-Str. 70, 17489, Greifswald, Germany

**Keywords:** GP-shortage, Delegation of GP-home visits, Rural regions, Ambulatory healthcare centre

## Abstract

**Background:**

The AGnES-concept (AGnES: GP-supporting, community-based, e-health-assisted, systemic intervention) was developed to support general practitioners (GPs) in undersupplied regions. The project aims to delegate GP-home visits to qualified AGnES-practice assistants, to increase the number of patients for whom medical care can be provided.

This paper focuses on the effect of delegating GP-home visits on the total number of patients treated. First, the theoretical number of additional patients treated by delegating home visits to AGnES-practice assistants was calculated. Second, actual changes in the number of patients in participating GP-practices were analyzed.

**Methods:**

The calculation of the theoretical increase in the number of patients was based on project data, data which were provided by the Association of Statutory Health Insurance Physicians, or which came from the literature.

Setting of the project was an ambulatory healthcare centre in the rural county Oberspreewald-Lausitz in the Federal State of Brandenburg, which employed six GPs, four of which participated in the AGnES project. The analysis of changes in the number of patients in the participating GP-practices was based on the practices’ reimbursement data.

**Results:**

The calculated mean capacity of AGnES-practice assistants was 1376.5 home visits/year. GPs perform on average 1200 home visits/year. Since home visits with an urgent medical reason cannot be delegated, we included only half the capacity of the AGnES-practice assistants in the analysis (corresponding to a 20 hour-work week). Considering all parameters in the calculation model, 360.1 GP-working hours/year can be saved. These GP-hours could be used to treat 170 additional patients/quarter year. In the four participating GP-practices the number of patients increased on average by 133 patients/quarter year during the project period, which corresponds to 78% of the theoretically possible number of patients.

**Conclusions:**

The empirical findings on the potential to increase the number of patients in GP-practices through delegation of tasks come close to the theoretical calculations. Differences between the calculated and the real values may be due to differences in the age and mortality distribution of the patients. The results indicate that a support system based on practice assistants can alleviate the consequences of GP-shortages in rural areas.

## Background

In Germany, general practitioners (GPs) have a leading role in primary care. Traditionally, other medical professions, including nurses, do not have a structural role in the organization of primary healthcare. GP-practice assistants mainly conduct non-medical tasks such as administration of appointments and coordination of the work flow of the practice. During the last years, however, the organization of GP-practices has changed towards a more team oriented approach involving the entire practice-staff. Practice assistants can now qualify for more specialized tasks (e.g. case management and other medical tasks [1,2]).

The Association of Statutory Health Insurance Physicians is responsible by law to guarantee an adequate number of physicians providing ambulatory care for each administrative district in Germany. The supply of ambulatory care is based on a fixed ratio between the numbers of physicians in practices and inhabitants of a defined region (usually at the county-level). This ratio is dependent on the speciality of the physician and differs somewhat between rural and urban regions. In Germany, a region is undersupplied with GPs if the ratio is below 75% of the calculated number of GPs for this region. Between 75% and 100%, the care situation in a region is categorized as imminently undersupplied [3].

About 20% of the GPs in Germany are 60 years of age or older and will retire in the next few years [4]. Few younger physicians are willing to start a private practice in a rural area, especially in the eastern part of Germany. In some regions, shortages in primary care are already existent or can be expected within the next years.

At the same time the number of elderly people living in the eastern part of Germany is rapidly increasing, due to demographic changes and migration to urban areas. This leads to a higher absolute number of age-associated chronic diseases and multimorbidity [5,6]. Elderly patients receive a significantly higher number of GP-home visits than younger patients (under 65-years old patients: on average 2.7 home visits/year, 65-79-years old patients: on average 6.3 home visits/year, 80+ year old patients: on average 8.5 home visits/year) [7].

However, the overall frequeny of GP-home visits decreases in Germany as well as in other European countries. A study of van den Berg et al. based on data of 183 Dutch GP-practices shows that the proportion of home visits decreased from 14.1% of all patient contacts in 1987 to 7.4% in 2001 [8]. The decrease has two main causes: fewer home visits involving children and involving patients with less urgent diagnoses. Obviously, a GP having to deal with a large number of patients has to weigh medical and non-medical factors (e.g. workload, availability in his practice) and will more often decide against conducting a home visit [8].

A qualitative study of Theile et al. among 24 GPs in Germany shows that GPs frequently conduct home visits with little or no medical indication. Home visits with vulnerable, elderly people, however, were often imperative [9]. Hence while the need for home visits will probably increase as a consequence of the growing number of older patients, the workload of GPs, particulary in rural areas, is expected to rise in the near future due to an undersupply of physicians.

The delegation of home visits to qualified GP-practice assistants could provide one possibility to support GPs. To examine the option of delegating GP-home visits, we developed and implemented the AGnES concept (AGnES = GP-supporting, community-based, e-health-assisted, systemic intervention) [10,11].

The main aim of the AGnES concept is to decrease the workload of GPs, especially in sparsely populated regions with an imminent or already existing undersupply of GPs. The concept enables the available GPs to provide care for more patients in a larger area [10].

All AGnES projects were conducted in “daily-routine-settings” with minimum intervention. The participating patients were selected by their treating GPs. The only inclusion criterion was that the patient, in the opinion of the GP, needed one or more home visits that could be delegated to a qualified practice assistant. The GP decided about the frequency and type of care provided during the home visit.

All together, 53 GPs and 40 AGnES-practice assistants participated in seven field studies between 2006 and 2008 in four federal states in Germany. The participating AGnES-practice assistants all received training specifically developed for this project [10,12].

In total, 11,228 home visits were carried out involving 1,430, mostly multimorbid patients with a mean age of 78.6 years (standard deviation: 10.7 years). 89% of the patients had limited or no mobility [10].

A previous analysis showed that the home visits conducted by the GPs significantly decreased, especially the number of medically urgent home visits. However, the overall rate of home visits (conducted by the GPs and the AGnES-practice assistants together) did not change significantly after implementation of the AGnES-concept. Thus, the additional possibility to provide home visits did not lead to a supply-driven increase of the total number of home visits [13].

The main aim of the AGnES projects was to support GPs in underserved regions and to enable GPs to increase their number of patients. To examine whether this aim was achieved, participating GPs were interviewed. About 90% of the participating GPs reported a reduced workload due to the AGnES-practice assistants. In an evaluation, the GPs stated that for more than 92% of the participating patients the quality of care provided by the AGnES-practice assistants was comparable to the usual care [10].

To obtain a more objective indication for the influence of the AGnES-concept on the workload of the GPs, we analyzed the development of the total number of patients treated in participating GP-practices in one of the project regions, the county Oberspreewald-Lausitz in the Federal State of Brandenburg. We choose this region for two reasons:

1. The county Oberspreewald-Lausitz has an imminent undersupply of GPs: in the year 2008 the degree of supply with GPs in this county was 95%. In 2011, this percentage decreased to 87%, indicating an imminent undersupply of primary medical care. A shortage of GPs is necessary to increase the number of patients not at the expense of other practices in the region.

2. The ambulatory healthcare centre in which all four participating GPs and the two non-participating GPs had their practices provided detailed data for the analysis.

The research questions are:

1. How many additional patients can a GP theoretically include in his practice under ideal conditions?

2. Did the implementation of the AGnES-concept actually result in an increase in the number of patients in the participating GP-practices in the county Oberspreewald-Lausitz in the Federal State of Brandenburg?

## Methods

The data needed to calculate the capacity of the AGnES-practice assistants was retrieved from the standardized project documentation which was identical in all AGnES projects (duration of the home visits, driving times, time needed for consultation with the GP and other activities). Information about the capacity of the GPs were taken both from published literature [14,15] and from data from the Associations of Statutory Health Insurance Physicians from Saxony and Mecklenburg-Western Pomerania.

The changes in the number of patients in GP-practices were analyzed using data of an ambulatory healthcare centre in the rural county Oberspreewald-Lausitz in the Federal State of Brandenburg (Eastern Germany). This healthcare centre participated in the AGnES-Brandenburg project from July 2006 until December 2008.

Six GPs had their practices in the ambulatory healthcare centre; four of these participated in the project and delegated home visits to AGnES-practice assistants. In total, three AGnES-practice assistants, all of them qualified nurses with extended professional experience, were working full-time in the AGnES-project. The GPs decided whether or not to delegate home visits to the AGnES-practice assistants.

The number of treated patients in the participating GP-practices was calculated based on reimbursement data for all patients of the GPs of the ambulatory healthcare centre, who were insured with one of the German statutory health insurances. This was true for more than 95% of the patients of the ambulatory healthcare centre. These data were available for each of the GP-practices of the ambulatory healthcare centre. To analyze the changes in the sizes of the practices, we used absolute numbers rather than relative changes. As the same relative increase in a small and a large practice involves a larger number of patients in the large practice, and since GPs have to treat real numbers of patients, we used the absolute numbers of patients.

All participating patients gave written informed consent. The study was approved by the ethics committee of the University Medicine Greifswald, Germany (Reg.-Nr. III UV 61/06).

## Results

To answer the first research question “How many additional patients can a GP theoretically include in his practice due to delegation under ideal conditions?” data of all seven AGnES-projects were used.

The main element of this analysis was the calculation of the mean capacity of AGnES-practice assistants. To calculate the real working time of the AGnES-practice assistants, 2008 was used as the reference year. This year had 104 Saturdays and Sundays, and 9 bank holidays. We assumed 31.1 vacation days, 7.3 days of sick leave [16] and 5.0 days for vocational training of the AGnES-practice assistants. Additionally, 5.0 days for administration and quality management within the GP-practice were subtracted from the total number of working days. In total, 203.6 net working days per year were calculated. Assuming a work-week of 40 hours, the average yearly working time of an AGnES-practice assistant amounts to 1,628.8 hours [17].

To calculate the yearly number of home visits that an AGnES-practice assistant can theoretically conduct, we took the mean length of all documented home visits within the AGnES-projects, which was 33.4 minutes (range 29–40.6 minutes). Additionally, we added:

driving time for each home visit (mean time 16.1 minutes; range 11.7-22.7 minutes), this includes associated drives, e.g. to the pharmacist or laboratory;

consultation with the GP (for each home visit: mean time 4.6 minutes; SD 3.2; range 3.6-6.7 minutes)

time for organization and documentation (for each home visit: mean time 17.3 minutes) [17].

In total, an average of 71 minutes was needed for each home visit. With reference to the calculated annual net working time of 1628.8 hours, the mean yearly capacity of an AGnES-practice assistant corresponds to 1,376.5 home visits.

GPs conduct, on average, 1,200 home visits a year [7]. A proportion of these home visits cannot be delegated to AGnES-practice assistants, e.g. due to a medical emergency or unclear situation of a patient. Hence the full capacity of an AGnES-practice assistant (1,376.5 home visits per year) exceeds by far the number of theoretically delegable home visits. Consequently, we assumed only a half-time position of the AGnES- practice assistants (which is a 20 hours working week) corresponding to 50% of their capacity (688 home visits per year).

The mean length of a home visit, conducted by a GP in Germany is 20.6 minutes (calculated form reimbursement data of the Associations of Statutory Health Insurance Physicians from Saxony and Mecklenburg-Western Pomerania). 12.4 minutes driving time are added (based on the driving times of the AGnES-practice assistants, without considering associated drives) [17] and 3.0 minutes of documentation, giving instructions to the practice team etc. In total, a GP needs 36.0 minutes for each home visit. Assuming that the AGnES-practice assistant can conduct 688 delegated GP-home visits per year, the GP would save 412.8 working hours a year. However, according to the data from the AGnES projects the GP invests on average 4.6 minutes to consult with the AGnES-practice assistant for each delegated home visit. Consequently, 52.8 hours have to be subtracted from the GP’s theoretical time gain. This leaves 360.1 GP working hours yearly that can be saved by implementing the AGnES-concept in a practice.

In this model we assume that the time a GP can save by delegating home visits to an AGnES-practice assistant is used to extend the number of treated patients per practice. In Germany, the mean time for each patient contact in a GP-practice is 7.6 minutes [18]. Assuming a mean additional time for documentation and administration of 3.0 minutes, every patient contact in a GP practice requires 10.6 minutes. Assuming an investment of all saved working time in additional patient contacts, the GP could conduct 2,038 additional patient contacts per year. On average, patients visit the GP-practice 2–3 times each quarter year [7]. This means the GP could extend his patient base by 170 additional patients per quarter year for each half time AGnES-practice assistant.

The second research question focused on the actual increase in number of patients in the participating GP-practices. We examined this question for the AGnES Brandenburg project which was conducted in the ambulatory healthcare centre with six GP-practices [10].

The four participating GPs delegated in total 4,454 home visits to 379 patients (mean age: 76.8 ± 12.7 years, range 21–100 years) to the AGnES-practice assistants.

**Figure 1:**
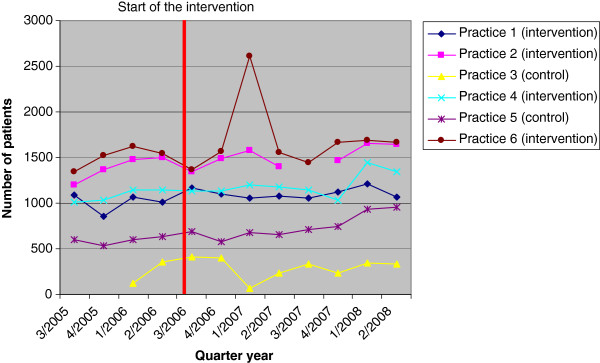
Numbers of patients in the GP-practices of the ambulatory healthcare centre, before and after the implementation of AGnES-Brandenburg.

Table 1 shows the number of patients of all GP-practices of the ambulatory healthcare centre per quarter. We considered four quarter years before and eight quarter years after the implementation of AGnES. Comparing the mean numbers of patients before and after the implementation of the AGnES-project, all six GP-practices increased the number of treated patients over the project period (Figure 1, Table 1).

**Table 1:** Numbers of patients in the GP-practices of the ambulatory healthcare centre, before and after the implementation of AGnES-Brandenburg

	Practice 1*	Practice 2*	**Practice 3**	Practice 4*	**Practice 5**	Practice 6*
***Before project:***						
**3. quarter 2005**	1084	1198	0^†^	1009	602	1346
**4. quarter 2005**	855	1363	0^†^	1032	530	1520
**1. quarter 2006**	1067	1482	123	1146	598	1620
**2. quarter 2006**	1015	1501	354	1145	628	1539
**Mean**	1005	1386	239	1083	590	1506
***During project:***						
**3. quarter 2006**	1168	1350	407	1130	684	1369
**4. quarter 2006**	1101	1484	402	1128	577	1571
**1. quarter 2007**	1052	1580	64	1201	674	2608
**2. quarter 2007**	1077	1396	235	1176	657	1556
**3. quarter 2007**	1057	8^†^	338	1142	710	1450
**4. quarter 2007**	1119	1464	236	1029	746	1665
**1. quarter 2008**	1216	1655	342	1443	932	1685
**2. quarter 2008**	1068	1645	332	1340	953	1665
**Mean**	1107	1511	295	1199	742	1696
**Difference**^**¥**^	102	125	56	116	152	190

* These practices participated in the AGnES-Brandenburg project.

^†^ During these quarter years, these practices were closed due to illness of the GPs. These quarters were not considered in the analyses.

^¥^ Difference between the means before and during the intervention.

In the four participating GP-practices, the mean number of patients before implementation of the AGnES-concept was 1,245 / quarter year. After implementation of the project, the mean number of patients increased to 1,378 patients / quarter year, an increase of 133, corresponding to about 78% of the theoretically possible increase of 170 patients / quarter year. In the two non-participating GP-practices, the mean number of patients before the start of the project was 415 / quarter year, during the project the mean number of patients increased to 519 / quarter year which is a plus of 104 patients / quarter year.

## Discussion and conclusion

The analyses described in this paper address the eligibility of the AGnES-concept for implementation into the regular health care system in Germany. The calculation of the number of additional patients a GP can treat in his practice due to delegation of home visits to AGnES practice-assistants was modelled, using parameters from the project documentation, and from the literature. In reality every GP-practice is different, regarding both the structure of the patient base and the practice organization. Our calculation therefore is meant to provide a numerical basis for the analysis, rather than to reflect the actual situation in the participating practices.

The results of the analysis are not transferable to any region but only to regions with equal preconditions, which are rural regions with an equal level of general practitioner care, an equal age distribution of the population, and an equal distribution of the morbidity of the population.

The number of patients varies over the quarter years by up to 100% in the established practices. In some instances, the study doctors provided explanations for this, e.g. sick leaves of the GP in the 3rd and 4th quarter year of 2005, and in the first quarter year of 2007 in practice 3, and in the 3rd quarter year of 2007 in practice 2. The increase of about 1000 patients in the first quarter year of 2007 in practice 6 was caused by the presence of an additional “GP in training” (Table 1). Other variations may be due to a variety of reasons including sick leave of other GPs in the region, and, since the region attracts many tourists, seasonal variations in the population size.

On average, a GP-practice in Germany provides medical care for about 900 patients / quarter year. The results of our analysis show that GPs with large practices quickly adapted to the possibility to delegate home visits, which is an indication of ready acceptance of the AGnES-concept by the most important target group.

One of the four participating practices (practice 6 in Table 1), with an increase of 190 patients during the project period, exceeds the calculated possible expansion of 170 patients. Since during the project three AGnES-practice assistants were available for the six GPs in the ambulatory healthcare centre but only worked for four of them, they were more flexible with their working time, which could be an explanation for the high increase of patients in practice 6.

Although the GP-practices participating in our study had already numbers of patients above the national average before the start of the AGnES-Brandenburg project, they increased the sizes of their practices on average by 133 patients / quarter year. With this increase they reached on average 78% of the possible expansion during the project period. Various factors could be responsible for not reaching the calculated maximum increase. The model uses mean values for the included variables that can be different in the analyzed GP-practices. Also regional structures with respect to the numbers of patients in the different sectors of the health care system may be important. The age and morbidity distribution of the population are major determinants of the need for medical care.

Other reasons may be the time required to adjust to this new concept, and its integration into the work-flow of the practice. Also the fact that the GPs did not have a guarantee of continuing the delegation-concept after finishing the project could possibly have kept them from exploiting this concept to its full extent.

Delegated home visits are not identical to GP-home visits. First of all, not all GP-home visits can be delegated. Home visits with an urgent medical reason usually have to be conducted by the GP himself. And secondly, the AGnES-practice assistants recorded more driving time needed for the home visits, because they had to drive to the pharmacist, the laboratory etc. This could be an indication for delegating particularly elaborate home visits. Furthermore, the AGnES-practice assistants had to complete some tasks which are normally delegated to the practice team, such as those belonging to the preparation for or finishing of a home visit, by themselves.

This study has several methodological limitations. Most importantly, participating practices self-selected themselves into the intervention or control group. Because the allocation did not follow a randomization procedure, we cannot rule out the possibility that other factors apart from the measured factors are partly or fully responsible for the observed effects. A descriptive comparison revealed some structural differences including size and working experience of the GPs in the non-participating practices. Hence, the non-participating practices do not fulfil the criteria for a control group and can not readily be used to estimate the secular trend of patient numbers for the four participating practices.

In theory, it would be easier for small practices than for larger practices to increase their numbers of patients. An interesting question is why the non-participating practices are small in a region with an imminent undersupply of GPs. The GP of practice 5 had just started her practice. This would explain why her number of patients increased steadily during the observation time and reached the average numbers of patients in the other practices only in the final observed quarter year. The GP of practice 3 was absent during the first two quarter years for medical reasons. After returning to work, she had inconstant and less working hours as the other GPs and was not interested to participate in the AGnES project.

Based on the positive results and high degree of acceptance of the AGnES-concept among GPs, practice assistants, and patients [10], in 2008 a legislation amendment was passed on the federal level that explicitly allowed the delegation of GP-home visits. Since 2009, the reimbursement of delegated home visits is possible in regions that are (imminently) underserved.

The results of this explorative analysis may support the hypothesis that an increase of the number of patients after implementing the AGnES-concept is possible. To provide more conclusive evidence, a prospective cluster-randomised study with a larger number of GP-practices in comparable regions would be preferable; alternatively reimbursement data of GPs, using the new legislation, could be analyzed to examine the effects of the AGnES-concept on the health care situation in selected underserved regions.

## Competing interests

The authors declare that they have no competing interests.

## Authors' contributions

NB, CM, SF, and WH participated in the design of the study. CM and RH participated in the acquisition of data. RH performed the modelling of the capacity of the AGnES-practice assistants. NB, RH and SB performed statistical analysis. All authors participated in the interpretation of the results. NB drafted the manuscript, all authors revised it critically. All authors read and approved the final manuscript.

## Author information

* Romy Heymann shared first authorship.

## Pre-publication history

The pre-publication history for this paper can be accessed here:

http://www.biomedcentral.com/1472-6963/12/355/prepub
